# On Medical Reform

**Published:** 1809-12

**Authors:** 


### *To the Editors of the Medical and Physical Journal.*

Gentlemen,

THE subject of Medical Reform has lately occupied a considerable share of the public attention, and we learn from Dr. Harrison's Circular Letter, that it will probably become more public in consequence of a bill, which it is intended to bring forward during the ensuing Session of Parliament.

The necessities of this case are so evident, that it would be wasting the time of yourselves and readers, to insist any farther upon them; and the general principles, according to which such a reform should be conducted, have been too extensively discussed, and are too well understood, to require any additional commentary in this place. It seems to be fully established, no less as a medical than as an axiom of political œconomy, that the division of labour tends to the perfection of art; and it is admitted as an incontrovertible truth, that the science of medicine requires no less than its sister sciences, such a cultivation of the intellectual and moral powers, as can result only from an

---

enlightened and liberal education. These principles have, moreover, been considered in their several relations with moderation and temper, which the circumstances of the case demanded, and which should ever characterize the proceedings of gentlemen and philosophers.

While, however, the necessity for these distinctions, and for these qualifications, has been particularly vindicated by the College, to what shall we attribute it, that in practice, these very gentlemen are daily contravening their own positions, and depreciating the valuable privileges which they have been so anxious to obtrude upon public notice, and which they are so justly desirous of maintaining? The inconsistency to which I allude, is the very frequent custom of recommending the untitled, and therefore irregular practitioners of medicine, in preference even to *the members of their own body*, who reside at the different watering places of this kingdom. As this fact can need no confirmation to those who have ever visited these resorts of the sick and the gay, it will be sufficient simply to state it in this place: neither will it be necessary to enlarge upon the injurious effects of this inconsistency, both to the general cause of reform, and to the particular question, which involves the legal and assumed rights of the College. We may, however, assert without fear of contradiction, that while the privileged advocates for reform thus undervalue the dignities of the profession, and the members of their own corporation, we can expert no radical change in the system of medical abuse; and must despair of seeing that respectability, which can only arise from a sense of mental superiority, and more extensive intellectual endowments, attached to a profession, which is now, by the courtesy of our country, placed at the very bottom of the lists of rank and fame.*

I am, &c.

VIATOR.

---

* See the table of precedence, published by the Herald's Office, in the Gentleman's Magazine for February or January last. It appears that a lieutenant in the navy takes precedence of a physician. At what period was this barbarous law enacted? And, upon what authority is it here asserted?

